# A Survey on Prevalence and Pathological Findings of Gallstones in Lori-Bakhtiari Sheep in Iran

**DOI:** 10.1100/2012/524607

**Published:** 2012-05-01

**Authors:** Afshin Raoofi, Alireza Rahmani Shahraki, Abdolrasool Namjoo, Hasan Momtaz

**Affiliations:** ^1^Department of Clinical Sciences, Faculty of Veterinary Medicine, University of Tehran, P.O. Box 14155-6453, Tehran, Iran; ^2^Club of Young Researchers, Islamic Azad University, Shahrekord Branch, P.O. Box 166, Shahrekord, Iran; ^3^Department of Clinical Sciences, Faculty of Veterinary Medicine, Islamic Azad University, Shahrekord Branch, P.O. Box 166, Shahrekord, Iran; ^4^Department of Microbiology, Faculty of Veterinary Medicine, Islamic Azad University, Shahrekord Branch, P.O. Box 166, Shahrekord, Iran

## Abstract

In a survey of 430 Lori-Bakhtiari sheep at a slaughterhouse in Iran, gallstones were found in the gallbladder of 7 sheep (1.6%). Biliary calculi were more frequent in adult and female sheep (*P* < 0.05). Chemical analysis of the gallstones revealed 6 sheep with pigment (bilirubin) stones and 1 sheep with cholesterol stones. Chemical composition of bile in these sheep was evaluated. Bacteriologic analysis of the bile in the affected sheep revealed bacteria (*Streptococcus* spp., *Klebsiella* spp., *Escherichia coli*, and *Salmonella* spp.) in 5 sheep. Microscopic examination of gallbladders revealed focal calcification, cystic glands, necrosis and atrophy of mucosal layer, edema, diffuse and focal infiltration of lymphocytes in submucosal layer, and hypertrophy of smooth muscles in sheep with gallstones. It was concluded that the prevalence of both types of gallstones in Lori-Bakhtiari sheep is low. Cholelithiasis can cause chronic inflammation of the gallbladder, but it is not likely to become clinically significant.

## 1. Introduction

The Lori-Bakhtiari sheep is one of the most common native breeds in the southwestern part of Iran, with more than 1.7 million head population, and the largest fat-tail size among all the breeds in Iran. Majority of the sheep population, are managed under a migratory system, utilizing the ranges as the major source of feed [1, 2]. Choleliths or gallstones are concretions of normally soluble components of bile. They occur infrequently in all the domestic species, but they are especially well described in ruminants [3]. There are two major types of gallstones (pigment and cholesterol), which seem to form due to distinctly different pathogenic mechanisms. Pigment stones are composed of large quantity of bile pigments, along with less amounts of cholesterol and calcium salts. Cholesterol stones can be almost pure cholesterol or mixtures of cholesterol and substances such as mucin [4]. Biliary stone formation begins with the precipitation or aggregation of normally soluble components of bile. Other mechanisms involved in the pathogenesis include ascariasis, ascending biliary infection or inflammation, biliary stasis, changes in bile composition, and presence of a foreign body [5]. Choleliths in the gallbladder usually do not become clinically significant unless they migrate and obstruct the extrahepatic bile ducts [3]. The pigment gallstones are more frequently detected in sheep (8.6%), and they are associated with high total bilirubin concentration in the bile [6]. Amazingly, the foetal sheep gallbladder is capable of containing concentrated bile that can produce gallstones. These stones are composed of calcium palmitate and pigment [7].

The clinical signs associated with cholelithiasis in the large animals are anorexia, weight loss, low milk yield, chronic intermittent diarrhea, recurrent attacks of sever abdominal pain, alimentary tract stasis, pain on percussion over the liver, pyrexia, jaundice, recumbency, depression, and coma. [8–10]. Hyperammonemic hepatic encephalopathy and photosensitization are other less common features of cholelithiasis. A subclinical presentation, caused by partial obstruction of the biliary tree, may be recognized only on postmortem examination [5]. Choleliths may be associated with chronic cholecystitis [3]. The lack of observational models for noninduced cholelithiasis in animals had contributed to the absence of epidemiologic studies, which have been done in human beings [6]. In view of the paucity of information on cholelithiasis in sheep, this paper reports the prevalence and composition of choleliths in a main breed of Iranian sheep. Bacteriologic analysis and histopathological findings were reviewed.

## 2. Materials and Methods

The study was carried out on 430 Lori-Bakhtiari sheep (279 males and 151 females) at Juneghan abattoir in Chaharmahal-Bakhtiari province of Iran from November 2009 to June 2010. Before slaughter, an antemortem examination was done on all sheep during which their age and gender were evaluated. A division of young (≤2 years, *n* = 297) and adult (>2 years, *n* = 133) sheep were done after inspection of their teeth. After slaughtering the sheep, the gallbladder was removed and the surface and some sections of the liver were examined macroscopically. A short incision was made in the gallbladder by use of a scissors. In sterile conditions, a sample of bile was collected by a swab for bacterial culture. Then, the gallbladder was opened and its contents passed through a filter (fourfold gauze) to collect the gallstones. A sample of bile was also collected from the sheep with gallstones for chemical analysis. Number, diameter, weight, color, consistency, and chemical composition of gallstones were determined. Gallstones were analyzed for the determination of cholesterol (CHOD-PAP), bilirubin (DCA), calcium (arsenazo), and phosphorous (phosphometric UV) concentrations. Measurements were performed by commercial kits (Pars Azmoon, Tehran, Iran) using an automated biochemical analyzer (Biotecnica, Roma, Italy) in accordance with the instructions by the manufacturers. Oxalate was determined (enzymatic method) with a commercial kit (Darman Kav, Tehran, Iran) using a spectrophotometer (Clima, Madrid, Spain). Bile samples were analyzed by the same methods using Hitachi 704 autoanalyzer (Tokyo, Japan). Tissue specimens from gallbladders with gallstones were fixed in neutral buffered 10% formalin, processed, and sectioned. Tissue sections were stained with hematoxylin-eosin and prepared for histopathological examination.

 The SPSS version 16 statistical package was used for data analysis. The chi-square and Fisher's exact tests were used for comparing the groups, and *P* < 0.05 was considered to be significant.

## 3. Results

Seven of the 430 sheep (1.6%) had 2 to 10 gallstones in their gallbladders. The clinical sings associated with cholelithiasis were not observed in the affected sheep. Necropsy examination revealed no visible findings in the liver of sheep with gallstones. Number, diameter, weight, color, consistency, and chemical composition of gallstones are shown in Tables 1 and 2. Chemical analysis of the gallstones revealed 6 sheep with pigment (bilirubin) stones and 1 sheep with cholesterol stones. Bacteriologic analysis of the bile in 7 sheep with gallstones revealed bacteria (*Streptococcus* spp., *Klebsiella* spp., *Escherichia coli,* and *Salmonella *spp.) in 5 sheep. Concentrations of total and direct bilirubin, cholesterol, calcium and phosphorous in the bile of the sheep with gallstones are shown in Table 3. The gallstones were more frequently found in adult than in young sheep (*P* < 0.05) and in female, rather than male (*P* < 0.05). Microscopic examination of gallbladders revealed focal calcification, cystic glands, necrosis and atrophy of mucosal layer, edema, diffuse and focal infiltration of lymphocytes in submucosal layer, and hypertrophy of smooth muscles in sheep with gallstones.

## 4. Discussion

The prevalence of gallstones in surveyed sheep was 1.6%, which was less than the prevalence in sheep (8.6%) reported by Cavallini et al. [6]. A study on prevalence of cholelithiasis in sheep indicated that 11.7% of sheep had gallstones [11]. Khaki [12] reported that the prevalence of gallstones in sheep and cattle slaughtered in Tehran abattoirs in Iran were 4.75% and 5.6%, respectively. A study on the gallbladders of 36 fetal sheep of gestational age 102–147 days revealed a 50% incidence of gallstones [7].

 In the present investigation, the gallstones were more frequently found in adult than young sheep. The risk of gallstones increases with age in human beings. An increase in age may be directly proportional with an increase in cholesterol secretion and saturation [13].

 The current study shows that the biliary calculi are more frequent in females compared with males. This is in agreement with what was reported by Cavallini et al. [6]. Female sex hormones are the obvious basis for this gender difference [13]. Progesterone and also probably estrogen impair gallbladder emptying and are associated with hypersecretion of cholesterol into bile. In addition, estrogen treatment reduces synthesis of bile acids. These proprecipitation factors peak during late pregnancy when the levels of these steroids hormones are at the highest [4]. However, the results of one study indicated higher frequency of gallstones in male sheep [11].

 In this study, most of the gallstones found in the sheep were pigment stones. This is in agreement with the results reported by Petruzzi et al. [11], Cavallini et al. [6], and Khaki [12]. All gallstones that they found in sheep and cattle were pigment type. These findings indicate that pigment cholelithiasis is more common in ruminants.

 Bacteriologic analysis of the bile in 5 of the 7 sheep with gallstones revealed bacteria (*Streptococcus* spp., *Klebsiella* spp., *Escherichia coli,* and *Salmonella* spp.). Isolation of bacteria from bile is common. In one study, bacteriologic analysis of the bile in 10 sheep with gallstones and 10 controls (without gallstones) revealed bacteria in 50% of the first group and 75% of the second group [6].

 Microscopic examination of gallbladders revealed focal calcification, cystic glands, necrosis and atrophy of mucosal layer, edema, diffuse and focal infiltration of lymphocytes in submucosal layer, and hypertrophy of smooth muscles. These findings indicate chronic cholecystitis which is most likely to be due to the mechanical irritation of the gallbladder by biliary calculi. Cholecystitis may occur as a result of bacterial infections such as salmonellosis. Other bacteria, either derived from the blood or ascended from the intestine, can cause acute or chronic cholecystitis. Chronic cholecystitis typically accompanies prolonged bacterial infection of the biliary tree or ongoing irritation from choleliths or parasites of the gallbladder [3].

 Based on the results of this study, the prevalence of both types of gallstones in Lori-Bakhtiari sheep is low. Although cholelithiasis can cause chronic inflammation of the gallbladder, it is not likely to become clinically significant.

## Figures and Tables

**Table 1:** Characteristics of the sheep and their gallstones.

				Gallstone			
Sheep number	Gender	Age (year)	Number	Diameter (mm)	Total weight (g)	Color	Consistency
1	Female	3	5	4	0.02	Green	Soft
2	Female	4	9	2	0.01	Brown	Semi hard
3	Female	4	10	1	0.03	Green	Soft
4	Male	3	6	1	0.05	Green	Soft
5	Male	4	2	4	0.02	Dark green	Soft
6	Female	1	8	1	0.01	Dark green	Soft
7	Female	1	9	1	0.02	Dark green	Soft

**Table 2:** Chemical composition of gallstones in the sheep.

	Gallstone				
Sheep number	Bilirubin (%)	Cholesterol (%)	Oxalate (%)	Calcium (%)	Phosphorous (%)
1	12	38	20	25	5
2	9	41	30	20	—
3	—	18	—	72	10
4	3	5	—	81	11
5	—	80	—	15	5
6	4	7	—	76	13
7	80	3	—	16	1

**Table 3:** Chemical composition of bile in the sheep with gallstones.

	Bile				
Sheep Number	Total bilirubin (mg/dL)	Direct bilirubin (mg/dL)	Cholesterol (mg/dL)	Calcium (mg/dL)	Phosphorous (mg/dL)
1	13.2	7.5	120	22	24
2	8.2	6	82	22	20
3	12	4.5	40	17	14
4	4	3	39	14	10
5	3	0.5	73	10	20
6	8.8	4	55	10	20
7	14	5	66	19	22
